# The Incidence and the Risk Factors for Pharyngocutaneous Fistula following Primary and Salvage Total Laryngectomy

**DOI:** 10.3390/cancers15082246

**Published:** 2023-04-12

**Authors:** Robert Šifrer, Primož Strojan, Ivana Tancer, Maja Dolenc, Simon Fugina, Sara Bitenc Zore, Aleksandar Aničin

**Affiliations:** 1 Department of Otorhinolaryngology and Cervicofacial Surgery, University Medical Centre Ljubljana, Zaloška 2, 1000 Ljubljana, Slovenia; 2 Faculty of Medicine, University of Ljubljana, Vrazov trg 2, 1000 Ljubljana, Slovenia; 3 Department of Radiation Oncology, Institute of Oncology, Zaloška 2, 1000 Ljubljana, Slovenia

**Keywords:** primary total laryngectomy, salvage total laryngectomy, fistula, surgical wound infection, piriform sinus, radiotherapy, chemoradiotherapy, dose of radiotherapy

## Abstract

**Simple Summary:**

The pharyngocutaneous fistula is the most common surgical complication following total laryngectomy. It is an abnormal canal connecting the pharyngeal canal and the skin of the neck. It has been associated with many undesirable consequences not only for the surgeon and patient but also from the economic point of view. However, many controversies still remain. Our aim was to analyse the incidence and risk factors in a large study set collected over a longer period of time. Four hundred twenty-two patients were included. The incidence was 23.9% and the risk factors included surgical wound infection, piriform sinus invasion, laryngectomy following previous (chemo)radiation and total radiation dose. With the prevention of the modifiable risk factors, we can lower the rate at which the fistula occurs. In this regard, we find surgical wound infection to be of the utmost importance as it can be directly influenced.

**Abstract:**

The pharyngocutaneous fistula (PCF) is the most common complication following a total laryngectomy (TL) with a wide range of incidence and various potential risk factors. The aim was to analyse the incidence and potential risk factors for PCF formation in a large study set collected over a longer period of time. In the retrospective study at the Department of Otorhinolaryngology and Cervicofacial Surgery of Ljubljana, 422 patients who were treated for head and neck cancer by TL between 2007 and 2020 were included. The comprehensive clinicopathologic data were collected including potential risk factors related to the patient, disease, surgical treatment and post-operative period for the development of fistulae. The patients were categorized into a group with the fistula (a study group) and one without it (a control group). The PCF then developed in 23.9% of patients. The incidence following a primary TL was 20.8% and 32.7% following salvage TL (*p* = 0.012). The results demonstrated that surgical wound infection, piriform sinus invasion, salvage TL, and total radiation dose were determined as independent risk factors for PCF formation. A diminishing surgical wound infection rate would contribute to a further reduction of the PCF rate.

## 1. Introduction

Pharyngocutaneous fistula (PCF) is one of the most common and challenging wound complications following a total laryngectomy (TL) [1,2,3]. It occurs when there is a failure in pharyngeal repair, resulting in a salivary leak [4]. Its appearance is associated with a delay in wound healing, swallowing and voice rehabilitation, the application of adjuvant treatment, and sometimes results in additional surgical treatment. All these consequences of PCF formation are associated with an increase in treatment costs and also negatively affect the prognosis by increasing the risk of distant metastasis [5]. With the prolonged and unintentional exposure of the soft tissues of the neck to the saliva from the neopharynx, PCF formation might result in the erosion of major arteries in the neck, finally causing a carotid blowout with consequent exsanguination [6]. 

Despite often being discussed in the pertinent literature, some controversies regarding PCF still exist, particularly concerning their incidence. It is probably due to the lack of clear definitions in this subject. For instance, PCF might be defined by the observation of saliva in the wound or a leak visible in the barium swallow test or biochemical detection of amylase in the discharge or finding blue dye in the surgical wound after the blue-dye swallowing test. In the literature, not only are a wide range of PCF incidence rates reported, from 0% [7] to 80% [8], but also a variety of potential risk factors. In general, the incidence of PCF particularly depends on the type of TL: after primary TL, the incidence is between 8 and 25% [1,4,9,10,11,12], and after salvage TL, it is considerably higher, reaching 14–57% [1,4,11,12,13,14].

According to a recent meta-analysis of 58 studies by Kim et al. from 2022 [15], the incidence of PCF is 21.69%. Another meta-analysis of 52 studies by Wang from 2020 reported the total PCF incidence of 21%, 15.2% after primary TL, and 32.6% after salvage TL [16]. The identified risk factors for the development of PCF in both studies were an age exceeding 60, a history of smoking, chronic obstructive pulmonary disease, coronary atherosclerosis, diabetes mellitus, previous radiotherapy (RT), previous tracheostomy, low preoperative albumins and haemoglobin, stages T3 and T4, supraglottic tumour site, additional pharyngectomy, salvage TL, primary tracheoesophageal puncture, and low postoperative haemoglobin and proteins [15,16]. Two older systematic reviews of 63 and 16 studies identified PCF in a primary setting at 15.5% and 11.6%, respectively, and after salvage TL, at 24.6% and 36.0%, respectively. Some additional risk factors were found to be statistically related to the development of PCF, such as a hypopharyngeal tumour site, previous chemoradiotherapy (CRT), blood transfusions, neck dissection, and positive surgical margins [4,11].

According to our experience gained from the analysis of 79 patients treated between 2004 and 2006, the overall incidence of PCF was 39.2%. In the primary TL group, the incidence was 32.1% (18/56), but was 56.5% (13/23) in the salvage TL group. Previous RT with or without concurrent chemotherapy, (i.e., (chemo)radiotherapy, (C)RT), the need for blood transfusion, and surgical wound infection (SWI) were recognized as independent risk factors in PCF creation [17]. Moreover, in a more recent group of 158 patients treated in our department between 2007 and 2012, the overall PCF rate was 30.4%, while it was 22.6% in the primary TL group (23/102) and 44.6% in the salvage TL group (25/56). The independent predictors for the PCF included a history of head and neck cancer (HNC), invasion of the piriform sinus, and SWI [18].

The observed variability in the incidence and risk factors reported in the literature likely originates in the inhomogeneity of research protocols and the small sample sizes of some of the studies. To overcome these disadvantages, we decided to analyse a large cohort of patients, using the same research protocols as in our previous reports with some additional potential risk factors. The research was performed separately for the patients after primary TL and salvage TL. Furthermore, all patients undergoing TL in our department with available data were included in the study. Thus, the aims of the present study were to analyse the incidence and potential risk factors for PCF formation in a large study set collected over a longer period of time.

## 2. Materials and Methods

Medical charts from consecutive patients treated for HNC with TL between January 2007 and December 2020 at the University Department of Otorhinolaryngology and Cervicofacial Surgery in Ljubljana, Slovenia were reviewed. After the completion of the diagnostic procedures, all patients were discussed, and their management was agreed upon by the Multidisciplinary Head and Neck Oncology Board. 

At surgery, the neopharynx was closed using T-shape technique by synthetic absorbable sutures made of polyglactin 910 or polyester poly (p-dioxanone). The sutures were either running or interrupted, which was left to the discretion of the surgeon. Primary closure was performed in the case that the width of the remaining pharyngeal mucosa was at least 4 cm; otherwise, the reconstruction was performed using one of the following reconstructive options: epiglottoplasty [19], pectoralis major flap (PM), supraclavicular artery island flap (SCAIF), radial forearm flap, and anterolateral thigh flap (ALT). Reconstruction with gastric pull-up was occasionally used for circumferential hypopharyngeal and cervical esophageal defects. All patients received nasogastric feeding tube for 2 weeks. Afterwards, the blue-dye swallowing test was performed, and if no blue colour was found in the healing wound, oral feeding was initiated. PCF was diagnosed in any period after surgery if saliva was clinically discovered in the wound. Subsequently, PCF was confirmed by the blue-dye swallowing test that revealed blue stain in the saliva in the neck.

The data associated with the patient, disease, treatment, and postoperative course were systematically collected. The patients were categorised into two groups, i.e., those who developed PCF (study group) and those who did not (control group). The incidence of PCF was calculated, and the groups were statistically compared according to potential risk factors for PCF development. The risk factors that were studied are presented in Tables S1–S4 in the Supplementary Materials.

Furthermore, the risk factors were analysed separately for patients following a primary TL and for those who had undergone previous (C)RT, i.e., salvage TL.

In the pertinent literature, the salvage TL is usually defined as a TL performed after failed non-surgical organ preservation protocol (following definite (C)RT) [12,20,21]. In contrast, the salvage TL could also be defined as a TL after any previous therapeutical intervention in the neck, including surgery (e.g., partial laryngectomy) and/or (C)RT or a combination of these [22]. In our study, the definition of the salvage TL is based on the former.

The statistical analyses were performed using the IBMI SPSS Statistics version 25 (Chicago, IL, USA). For comparative analyses, the chi-square test, *t*-test, and Mann–Whitney U test were used. To determine the independent predictive values for the PCF formation of different potential risk factors under evaluation, binary logistic regression analysis was undertaken. Only those factors that proved to be significant in the univariate analysis were included. All statistical tests were two-sided and *p*-values below 0.05 were considered statistically significant. The results are presented as an odds ratio with a 95% confidence interval.

## 3. Results

A total of 422 patients were included in the study. The primary tumour sites included the larynx in 273 patients (64.7%), hypopharynx in 139 patients (33.0%), oropharynx in 8 patients (1.9%), and the oral cavity and thyroid gland in 1 patient (0.2%) each. The mean age of said patients was 64 years (range 37–89), and 383 (90.8%) of these were male.

TNM staging was performed according to the 8th edition of the TNM classification of malignant tumours. The primary tumour classification was pT1 in 11 patients (2.6%), pT2 in 46 patients (11.0%), pT3 in 206 patients (49.3%), and pT4a in 155 patients (37.1%). The neck was staged pN0 in 207 patients (49.2%), pN1 in 31 patients (7.4%), pN2a in 24 patients (5.7%), pN2b in 29 patients (6.9%), pN2c in 12 patients (2.9%), and pN3b in 118 patients (28.0%). The T and N stages remained unknown in 4 and 1 patient, respectively. All patients were free from distant metastases.

Most of the patients suffered from one or more concurrent diseases. In particular, 226 (53.6%) had cardiovascular diseases, 111 (26.3%) had gastrointestinal tract diseases, 97 (23.0%) had respiratory tract diseases, 70 (16.6%) had hypercholesterolemia, 63 (14.9%) had central nervous system diseases, 50 (11.8%) had diabetes mellitus, and 117 patients (27.7%) suffered from other diseases. Further details on the comorbidities and their particular treatments were not collected in the data, apart from previous head and neck cancer, which is detailed below. Three hundred and forty patients (84.5%) were smokers, either as active or exsmokers (that ceased smoking at least 2 years prior to TL). Regular drinking of alcoholic beverages was confirmed by 222 patients (55.9%); the exact quantity, however, was not determined in the study. A history of previous cancer (any site) was registered in 154 patients (36.5%), and previous HNC (including mucosal, cutaneous, thyroid, and salivary tumours) was reported in 122 patients (28.9%). 

The origin of previous HNC included the larynx in 84 patients, (68.9%), pharynx in 20 patients (16.5%), oral cavity in 7 patients (5.7%), and the oesophagus and thyroid gland had 2 cases (1.6%) each. Multiple tumour sites were observed in 7 patients (5.7%). As a part of the treatment of previous HNC, 69 patients (16.4%) were treated using RT, 41 (9.7%) using CRT, and 51 (12.1%) were treated via surgical resection of the tumour, 35/51 in combination with adjuvant (chemo)radiation. A primary TL was performed on 312 patients (73.9%), whereas salvage TL was performed on 110 patients (26.1%).

PCF developed in 101 of 422 patients (23.9%). Its incidence after primary TL was 20.8% (65/312) and 32.7% (36/110) after salvage TL. The difference was statistically significant (*p* = 0.012, chi-square test). The PCF appeared, on average, on the 12th day after TL (range 2–45), on the 12th day after primary TL (range 2–31), and on the 14th day after salvage TL (range 2–45).

The statistically significant risk factors for the development of PCF as determined by univariate analysis and the influence of prior interventions in head and neck for all patients are detailed in Table 1. However, all risk factors that were studied are outlined in the Tables S1–S4 that can be found in the Supplementary Materials.

The results of a binary logistic regression analysis of factors proved significant upon univariate analysis for all patients and are shown in Table 2. Only SWI, the invasion of piriform sinus, and salvage TL were identified as independent predictors for the development of PCF.

The risk factors for the development of PCF after primary TL and after salvage TL (that were statistically significant in both univariate analysis and binary logistic regression) can be seen in Table 3 and Table 4.

SWI was identified as an independent risk factor in both types of TL. In addition, the invasion of the piriform sinus proved to be an independent risk factor for the development of PCF in primary TL, while the salvage group, alternatively, showed RT dose as a risk factor. The independent risk factors for the development of PCF after primary and salvage TL are reported in Table 4.

## 4. Discussion

The incidence of PCF in the present series was 23.9%. Specifically, 20.8% after primary TL and 32.7% after salvage TL. The results further demonstrated that salvage TL, invasion of the piriform sinus, and SWI were independent predictors for the occurrence of PCF. In terms of salvage TL, besides SWE, the dose of the RT was an additional independent risk factor for PCF.

The incidence of PCF as well as its rates in the primary and salvage setting are inconsistently reported in the literature. After primary TL, the rate of PCF was found to range between 8 and 25% [1,4,9,10,11,12], and the incidence of 20.8%, after primary TL, in our series is within the expected range. Similarly, the reported incidence of PCF after salvage TL was between 20 and 40% [1,4,11,13,14], but a wider range of 14–57% was additionally stated [12]. Our analysis revealed an incidence of PCF of 32.7% after a salvage procedure, which is in agreement with the data available in the literature. However, the wide ranges reported by different authors indicate that the issue of PCF after TL is yet to be solved.

The first and the strongest independent risk factor for PCF in our series was SWI (*p* < 0.001). For the purpose of the study, we defined SWI as the erythema, oedema, and induration of the suture line and surrounding skin, corresponding to grade 3 in the classification scale by Tabet and Johnson [23]. In general, the surgical wound after TL is classified as clean-contaminated because, during the surgery, the hollow organ (pharynx) is breached and the tissues are exposed to the bacterial flora of the upper aerodigestive tract [23,24]. In the literature, the reported SWI rates after TL range from 10% [25] to 44% [26], and in our study, the incidence of SWI was 28.7%. SWI is a fearsome complication of all surgical procedures: specifically, in the case of TL, it can have devastating effects, including PCF formation [18,27].

One of the greatest advances in head and neck surgery is the introduction of antibiotic prophylaxis, resulting in the reduction of post-TL sepsis from 87% [28] to 10% [29]. SWIs in head and neck surgery are polymicrobial, so the antibiotic coverage should include aerobic, anaerobic, and Gram-negative bacteria. The type of antibiotic used varies between countries and institutions and often reflects the surgeons’ training and practices [24]. 

In our department, clindamycin and amoxicillin/clavulanate (both intravenously) are mainly used as prophylactic antibiotic regimens in patients undergoing clean-contaminated surgery for HNC. The former was administered in 33.4% and the latter in 63.5% of cases. Significantly, we terminated the use of clindamycin in favour of amoxicillin/clavulanate in 2011, after the decision was made to diminish the rate of SWI. As illustrated in Table 1, 49.5% of patients were treated with amoxicillin/clavulanate in the PCF group and 67.9% in control group. The respective results for clindamycin are 48.5% and 28.7%. As the difference in the use of specific antibiotics attained statistical significance in univariate analysis (*p* = 0.025), it provides solid evidence that amoxicillin/clavulanate is more efficient. Recent publications have even stirred the debate that clindamycin is, in fact, a risk factor for SWI due to its insufficient Gram-negative coverage and bacteriostatic (not bacteriocidic) mechanism of action [30]. In fact, this was also indirectly confirmed by our results. Notwithstanding, we believe that the introduction of amoxicillin/clavulanate prophylactic antibiotic regimen first decreased the overall rate of SWI (from 36.7% to 28.7%), followed by a reduction in the rate of PCF (from 30.4% to 23.9%) [18]. 

The duration of antibiotic prophylaxis is controversial: it usually lasts 3 days, although it may vary from 24 h to 7 days postoperatively [27]. The suggested length is a maximum of 24 h postsurgery, even for the patients with risk factors for SWI, such as previous (C)RT, complex resection with reconstruction, revision surgery, and salvage surgery [24]. The prolonged application of prophylaxis may lead to resistance and the increased incidence of infection and should be avoided [24]. However, the recommended length is often not adhered to, as was also the case in our series (Table 1). The patients with primary TL were treated with antibiotics on average for 7 days, whereas the patients with salvage TL for 8 days.

The second independent risk factor for PCF identified in our study was an invasion of the piriform sinus (*p* < 0.001). Similar results were reported by Michael et al. [31] and Mendelsohn et al. [32]. However, in recent meta-analyses, a piriform sinus invasion was recognized as a significant risk factor only by Dedivitis et al., increasing the risk of PCF by 9% [4]. Two other meta-analyses did not analyse the hypopharyngeal site as a potential risk factor at all [11,16] and the same applies to the study of Kim et al. Instead, these authors found an association between PCF and TL in patients undergoing pharyngectomy [15]. As pharyngectomy is performed with TL only in advanced hypopharyngeal cancer cases, we could speculate that the hypopharyngeal infiltration was also recognized as a predisposing factor for PCF in Kim’s meta-analysis. Furthermore, Wang et al. and Liang et al. identified the supraglottic tumour site as a risk factor for PCF. This is also indicatory because in supraglottic and hypopharyngeal cancer cases, the surgical resection must be more extensive in comparison to simple TL. When parts of oropharyngeal and/or hypopharyngeal mucosa are included in a surgical resection, less tissue remains available for the primary closure of the neopharynx. If it is performed under tension or even without the appropriate inversion of the mucosa into the lumen of the neopharyngeal canal, the wound is more prone to break down and dehiscence which both raise the risk for PCF [18,31].

In the present study, salvage TL (i.e., after (C)RT) was recognized as an additional independent predictor for the development of PCF (*p* = 0.001), as was the case in recent meta-analyses [15,16]. Previous RT and CRT are the most frequently studied risk factors [16]. RT induces fibrosis, obliterative endarteritis, impaired leukocyte migration, and the disorder of local blood circulation resulting in a decrease in the local skin and mucosa’s healing ability [1,16,18,33].

Furthermore, older reports suggest that high cumulative radiation dose, large RT fields, and higher daily fractions are contributing factors [32,34]. For example, Mendelsohn and Bridger found that a prior RT dose above 50 Gy is the most significant risk factor for PCF [32]. Similarly, Johansen et al. revealed a pronounced increase in the frequency of PCF with larger doses of RT, especially in the range between 68 and 72 Gy when compared to lower doses [34]. Grau discovered that a total radiation dose of 66 Gy and larger radiation fields, especially more than 169 cm^2^, are significantly related to PCF [35], but only in univariate analysis. In addition, Johansen et al. also found that fields larger than 50 cm^2^ in size induce an increase for PCF formation [34]. Some other authors challenged these findings [36]. Based on our results, we agree that the dose of RT plays a role in PCF formation (*p* = 0.036), while the size of the fields was not studied in our research. Nevertheless, if previously irradiated patients develop PCF, these are often extensive, persistent, and usually require surgical intervention [1,37,38]. 

As the concurrent chemotherapy increases the local effect of RT on tumours, the toxic effects of CRT on the surrounding normal tissues are also expected to be potentiated. Surprisingly, CRT appeared as a significant factor only in one meta-analysis reported by Dedivitis et al. The same authors calculated that RT increases the absolute risk for PCF formation by 8% and CRT by 11% [4]. The significant relationship between the CRT and PCF rate was also observed in our study, though on the univariate analysis only (16.8% in PCF group vs. 7.5% in control group, *p* = 0.006).

The literature’s data on the influence of previous surgical interventions due to HNC (and not for non-malignant diseases) on PCF formation are scarce. In our research, we did not find any relationship between prior surgery and PCF. On the contrary, Natvig et al. reported an increased risk for PCF after previous neck dissection (38% vs. 12%) [39].

It is unexpected that previous (C)RT was recognized as an independent predictive factor for PCF while any prior therapeutical intervention (including (C)RT or surgery) was not. In fact, RT, and particularly CRT, leads to extensive and diffused tissue change in terms of fibrosis and obliterative endarteritis and results in disorders of local blood circulation and tissue hypoxia, hindering the healing of the surgical wound considerably. The tissue damage after (C)RT is obviously more pronounced and detrimental than scarring following surgical intervention.

As compared to our historical experience, in the present study, we recorded a significant reduction in the PCF rate (from 30.4% during 2007–2012 to 23.9% between 2007 and 2020) [18]. From our point of view, the main reason was the reduction of SWI which was achieved primarily through the modification of the antibiotic prophylaxis. In addition, the meticulous and personal care of the surgical wound by the surgeons was of utmost importance. This includes daily wound monitoring and the use of (bactericidic) alcohol solution during the changing of the bandages and a policy of early treatment of SWI. Other reasons could potentially include improved anaesthesiologic and medical preparation for extensive oncologic surgery and the optimization of nutritional support. However, these measures were not studied within the scope of the present research.

## 5. Conclusions

PCF remains one of the possible complications of TL, and despite being thoroughly studied by myriads of authors, their rates are highly variable and risk factors continue to be elusive. The total incidence in our series was 23.9%: specifically, 20.8% after primary TL and 32.7% after salvage TL. SWI, piriform sinus invasion, salvage TL, and total radiation dose were determined as independent risk factors for PCF formation. As the location of the primary tumour and eventual prior treatments in the head and neck region cannot be influenced, we must be aware of factor(s) that we can modify. Thus, a diminishing SWI rate would contribute to further reduction of the PCF rate. It is of paramount importance that we are aware of all possible risk factors in order to know how to take appropriate action before, during, and after surgery in each individual patient.

## Figures and Tables

**Table 1:** Statistically significant risk factors for PCF by univariate analysis and the influence of previous treatment interventions and RT dose on PCF for all patients; regional/microvascular reconstruction includes pectoralis major flap (PM), supraclavicular artery island flap (SCAIF), radial forearm flap, anterolateral thigh flap (ALT), and gastric pull-up (^a^ *T*-test, ^b^ Chi-square test, ^c^ Mann–Whitney U test).

Risk Factor	Overall	PCF	Without PCF	*p* Value
All Patients	422	101	321	
Age (years), mean, range	63.79 (37–89)	62.06 (40–85)	64.34 (37–89)	0.038 ^a^
Comorbidity				
Previous cancer (any site)	154 (36.5%)	46 (45.5%)	108 (33.6%)	0.030 ^b^
Previous HNC	122 (28.9%)	40 (39.6%)	82 (25.5%)	0.007 ^b^
Treatment of previous HNC				
Surgery	51 (12.1%)	14 (13.9%)	37 (11.5%)	0.530 ^b^
RT	69 (16.4%)	19 (18.8%)	50 (15.6%)	0.443 ^b^
CRT	41 (9.7%)	17 (16.8%)	24 (7.5%)	0.006 ^b^
(C)RT	110 (26.1%)	36 (35.6%)	74 (23.1%)	0.012 ^b^
Surgery or RT or CRT	124 (29.4%)	40 (39.6%)	84 (26.2%)	0.010 ^b^
Dose of RT (Gy), median, range	67 (15.75–74)	70 (56–70)	64 (15.75–74)	0.002 ^c^
Invasion of subsites				
Piriform sinus	166 (39.3%)	50 (49.5%)	116 (36.1%)	0.016 ^b^
Retrocricoid area	72 (17.1%)	26 (25.7%)	46 (14.3%)	0.008 ^b^
Posterior wall of hypopharynx	31 (7.3%)	14 (13.9%)	17 (5.3%)	0.004 ^b^
Hypopharynx—median line	53 (12.6%)	19 (18.8%)	34 (10.6%)	0.038 ^b^
Hypopharynx—bilaterally	25 (5.9%)	11 (10.9%)	14 (4.4%)	0.015 ^b^
Primary site				
Larynx	273 (64.7%)	57 (56.4%)	216 (67.3%)	0.049 ^b^
Hypopharynx	139 (32.9%)	40 (39.6%)	99 (30.8%)	
Oropharynx	8 (1.9%)	2 (2.0%)	6 (1.9%)	
Oral cavity	1 (0.2%)	1 (1.0%)	0 (0)	
Thyroid gland	1 (0.2%)	1 (1.0%)	0 (0)	
Reconstruction				
Epiglottoplasty	54 (12.8%)	21 (20.8%)	33 (10.3%)	0.006 ^b^
Regional/microvascular	50 (11.8%)	20 (19.8%)	30 (9.3%)	0.005 ^b^
Type of regional/microvascular reconstruction				0.009 ^b^
PM	37 (8.8%)	13 (12.9%)	24 (7.5%)	
SCAIF	2 (0.5%)	1 (1.0%)	1 (0.3%)	
Radial forearm	3 (0.7%)	2 (2.0%)	1 (0.3%)	
Radial forearm-hybrid	3 (0.7%)	3 (3.0%)	0 (0)	
ALT	4 (0.9%)	1 (1.0%)	3 (0.9%)	
Gastric pull-up	1 (0.2%)	0 (0)	1 (0.3%)	
Regional/microvascular	50 (11.8%)	20 (19.8%)	30 (9.3%)	0.005 ^b^
Blood transfusion	99 (23.5%)	31 (30.7%)	68 (21.2%)	0.049 ^b^
Antibiotic prophylaxis	421 (99.8%)	101 (100.0%)	320 (99.7%)	0.578 ^b^
Type of Prophylactic antibiotic				0.025 ^b^
None	1 (0.2%)	0 (0)	1 (0.3%)	
amoxicillin/clavulanate	268 (63.5%)	50 (49,5%)	218 (67.9%)	
clindamycin	141 (33.4%)	49 (48.5%)	92 (28.7%)	
other regimens	12 (2.9%)	2 (2.0%)	10 (3.1%)	
Duration of antibiotic prophylaxis (days), mean, range	7.57 (0–48)	9.28 (1–48)	7.03 (0–35)	<0.001 ^a^
Surgical wound infection	121 (28.7%)	76 (75.2%)	45 (14.0%)	<0.001 ^b^

**Table 2:** A binary logistic regression model for all patients.

Risk Factors	Odds Ratio	95% CI	*p* Value
Surgical wound infection	22.61	12.44–41.08	<0.001
Invasion of piriform sinus	3.07	1.67–5.63	<0.001
Salvage TL	2.85	1.50–5.39	0.001

**Table 3:** Statistically significant risk factors for PCF by univariate analysis on PCF for patients after primary and after salvage TL (^b^ Chi-square test, ^c^ Mann–Whitney U test).

Risk Factor	Overall	PCF	Without PCF	*p* Value
Primary TL	312	65	247	
Invasion of piriform sinus	140	40 (61.5%)	100 (40.5%)	0.002 ^b^
Surgical wound infection	86	51 (78.5%)	35 (14.2%)	<0.001 ^b^
Salvage TL (after (C)RT)	110	36	74	
Dose of RT (Gy), median, range	65 (15.75–74)	70 (55–70)	64 (15.75–74)	0.002 ^c^
Surgical wound infection	35	25 (69.4%)	10 (13.5%)	<0.001 ^b^

**Table 4:** A binary logistic regression model for patients following primary and salvage TL.

Risk Factors	Odds Ratio	95% CI	*p* Value
Primary TL			
Surgical wound infection	25.32	12.17–52.67	<0.001
Invasion of piriform sinus	3.24	1.58–6.65	0.001
Salvage TL			
Surgical wound infection	16.08	5.62–46.00	<0.001
Dose of RT	1.18	1.01–1.37	0.036

## Data Availability

The data can be shared up on request.

## Supplementary Materials

The following supporting information can be downloaded at: https://www.mdpi.com/article/10.3390/cancers15082246/s1, Table S1: Potential risk factors for PCF associated with the patient by univariate analysis for all patients (^a^ *T*-test, ^b^ Chi square test, ^c^ Mann-Whitney U test); Table S2: Potential risk factors for PCF associated with the disease by univariate analysis for all patients (^a^ *T*-test, ^b^ Chi square test, ^c^ Mann-Whitney U test); Table S3: Potential risk factors for PCF associated with the surgical treatment (TL) by univariate analysis for all patients (^a^ *T*-test, ^b^ Chi square test, ^c^ Mann-Whitney U test); Table S4: Potential risk factors for PCF associated with the postoperative course by univariate analysis for all patients (^a^ *T*-test, ^b^ Chi square test, ^c^ Mann-Whitney U test).
