# Supplementary material for: The Incidence and the Risk Factors for Pharyngocutaneous Fistula following Primary and Salvage Total Laryngectomy
Source: Cancers (Basel). 2023 Apr 12;15(8):2246. doi: 10.3390/cancers15082246 (PMC10136624; doi:10.3390/cancers15082246)
Supplement: Supplementary file 1 [file cancers-15-02246-s001.zip › cancers-2275842-supplementary.pdf]

Risk factor associated with the patient	Overall	PCF	Without PCF	P value
**All patients**	**422**	**101**	**321**	
**Age** (years), mean, range	63.79 (37-89)	62.06 (40-85)	64.34 (37-89)	0.038 [a]
Sex				0.893 [b]
Male	383	91 (90.1%)	292 (91.0%)	
Female	39	10 (9.9%)	29 (9.0%)	
Comorbidity				
Cardiovascular	226 (53.6%)	61 (60.4%)	165 (51.4%)	0.114 [b]
Gastrointestinal	111 (26.3%)	20 (19.8%)	91 (28.3%)	0.089 [b]
Respiratory	97 (23.0%)	18 (17.8%)	79 (24.6%)	0.157 [b]
Hypercholesterolemia	70 (16.6%)	15 (14.9%)	55 /17.1%)	0.591 [b]
Central nervous system	63 (14.9%)	20 (19.8%)	43 (13.4%)	0.0115 [b]
Diabetes mellitus	50 (11.8%)	15 (14.9%)	35 (10.9%)	0.284 [b]
Other	117 (27.7%)	23 (22.8%)	94 (29.3%)	0.202 [b]
Previous cancer (any site)	154 (36.5%)	46 (45.5%)	108 (33.6%)	0.030 [b]
Previous HNC	122 (28.9%)	40 (39.6%)	82 (25.5%)	0.010 [b]
Weight loss				
Weight loss reported	105 (38%)	23 (36.5%)	82 (38.5%)	0.775 [b]
Amount of kg loss (kg), median, range	6.5 (0-43)	6 (0-43)	5 (1.5-28)	0.094 [c]
***Abuse** of*				
Tobacco	340 (85.4%)	81 (88.0%)	259 (84.6%)	0.417 [b]
Alcohol	222 (55.9%)	51 (56%)	171 (55.9%)	0.987 [b]
Preoperative laboratory values				
Haemoglobin (g/L), mean, range	132.71, (85-198)	130.96 (85-198)	133.24, (92-172)	0.232 [a]
Albumin	41.04, (26-72)	40.93 (28-50)	41.07, (26-72)	0.798 [a]
Proteins	68.41, (45-85)	68.15 (51-81)	68.49, (45-85)	0.663 [a]
Location of previous HNC				0.431 [b]
Larynx	84 (68.9%)	29 (72.5%)	55 (67.1%)	
Pharynx	20 (16.4%)	5 (12.5%)	15 (18.3%)	
Esophagus	2 (1.6%)	0 (0)	2 (2.4%)	
Oral cavity	7 (5.7%)	1 (2.5%)	6 (7.3%)	
Thyroid gland	2 (1.6%)	1 (2.5%)	1 (1.3%)	
More primary sites	7 (5.7%)	4 (10.0%)	3 (3.7%)	
Treatment of previous HNC				
Surgery	51 (12.1%)	14 (13.9%)	37 (11.5%)	0.530 [b]
RT	69 (16.4%)	19 (18.8%)	50 (15.6%)	0.443 [b]
CRT	41 (9.7%)	17 (16.8%)	24 (7.5%)	0.006 [b]
(C)RT	110 (26.1 %)	36 (35.6%)	74 (23.1%)	0.012 [b]
Surgery or RT or CRT	124 (29.4%)	40 (39.6%)	84 (26.2%)	0.010 [b]

Dose of RT (Gy), median, range	67 (15.75-74)	70 (56-70)	64 (15.75-74)	0.002 [c]
Interval (previous surgery-TL)				0.576 [c]
Interval (previous RT-TL)				0.397 [c]
Type of previous surgery				0.355 [b]
Vertical hemi-laryngectomy	7 (13.7%)	0 (0)	7 (18.9%)	
Endoscopic cordectomy	11 (21.6%)	6 (42.9%)	5 (13.5%)	
Extended tonsillectomy	5 (9.8%)	2 (14.3%)	3 (8.1%)	
Resection of oropharyngeal cancer	5 (9.8%)	2 (14.3%)	3 (8.2%)	
Thyreofissure	3 (5.9%)	1 (7.1%)	2 (5.4%)	
Resection of oral cavity cancer or Osteoradionecrosis surgery	8 (15.7%)	2 (14.3%)	6 (16.2%)	
Supraglottic laryngectomy	5 (9.8%)	0 (0)	5 (13.5%)	
Supracricoid laryngectomy	1 (2.0%)	0 (0)	1 (2.7%)	
Partial Hypopharyngectomy	1 (2.0%)	0 (0)	1 (2.7%)	
Supratracheal larygectomy	2 (3.9%)	0 (0)	2 (5.4%)	
Surgery of parapharyngeal abscess	3 (5.9%)	1 (7.1%)	2 (5.4%)	
ASA score				0.718 [b]
II	29 (14.6%)	5 (11.6%)	24 (15.4%)	
III	163 (81.9%)	37 (86.0%)	126 (80.8%)	
IV	7 (3.5%)	1 (2.3%)	6 (3.8%)	

Table S1. Potential risk factors for PCF associated with the patient by univariate analysis for all patients ([a]T-test, [b]Chi square test, [c]Mann-Whitney U test)

Risk factor associated with the disease	Overall	PCF	Without PCF	P value
**All patients**	**422**	**101**	**321**	
Primary site				0.049 [b]
Larynx	273 (64.7%)	57 (56.4%)	216 (67.3%)	
Hypopharynx	139 (32.9%)	40 (39.6%)	99 (30.8%)	
Oropharynx	8 (1.9%)	2 (2.0%)	6 (1.9%)	
Oral cavity	1 (0.2%)	1 (1.0%)	0 (0)	
Thyroid gland	1 (0.2%)	1 (1.0%)	0 (0)	
Invasion of subsites				
Glottis	273 (64.7%)	62 (61.4%)	211 (65.7%)	0.425 [b]
Supraglottis	299 (70.9%)	64 (63.4%)	235 (73.2%)	0.058 [b]
Subglottis	154 (36.5%)	31 (30.7%)	123 (38.4%)	0.165 [b]
Larynx – median line	226 (53.6%)	41 (40.6%)	185 (57.6%)	0.003 [b]
Larynx - bilaterally	167 (39.6%)	30 (29.7%)	137 (42.7%)	0.020 [b]
Piriform sinus	166 (39.3%)	50 (49.5%)	116 (36.1%)	0.016 [b]
Retrocricoid area	72 (17.1%)	26 (25.7%)	46 (14.3%)	0.008 [b]
Posterior wall of hypopharynx	31 (7.3%)	14 (13.9%)	17 (5.3%)	0.004 [b]
Hypopharynx – median line	53 (12.6%)	19 (18.8%)	34 (10.6%)	0.038 [b]
Hypopharynx – bilaterally	25 (5.9%)	11 (10.9%)	14 (4.4%)	0.015 [b]
Oropharynx	66 (15.6%)	22 (21.8%)	44 (13.7%)	0.051 [b]
Oropharynx – median line	22 (5.2%)	4 (4.0%)	18 (5.6%)	0.516 [b]
Oropharynx - bilaterally	22 (5.2%)	4 (4.0%)	18 (5.6%)	0.516 [b]
Histologic diagnosis				0.479 [b]
SCC	396 (93.8%)	95 (94.1%)	301 (93.8%)	
Sarcoma	3 (0.7%)	0	1 (0.3%)	
Medullary carcinoma	1 (0.2%)	1 (1.0%)	0 (0)	
Verrucous + SCC	2 (0.5%)	0 (0)	2 (0.6%)	
Spindle cell + SCC	6 (1.4%)	2 (2.0%)	4 (1.2%)	
Mucoepidermoid carcinoma	2 (0.5%)	0 (0)	2 (0.6%)	
Adenocarcinoma	1 (0.2%)	1 (1.0%)	0 (0)	
Papillary SCC	2 (0.5%)	1 (1.0%)	1 (0.3%)	
Basaloid carcinoma	7 (1.7%)	1 (1.0%)	6 (1.9%)	
Melanoma	1 (0.2%)	0 (0)	1 (0.3%)	
Adenoid cystic carcinoma	1 (0.2%)	0 (0)	1 (0.3%)	
Histologic grade				0.544 [b]
I	3 (0.7%)	1 (1.0%)	2 (0.7%)	
I-II	47 (11.7%)	11 (11.2%)	36 (11.9%)	
II	213 (53.1%)	54 (55.1%)	159 (52.5%)	
II-III	113 (28.2%)	23 (23.5%)	90 (29.7%)	
III	25 (6.2%)	9 (9.2%)	16 (5.3%)	
T stage				0.837 [b]
pT1	11 (2.6%)	2 (2.0%)	9 (2.8%)	

pT2	46 (11.0%)	12 (12.0%)	34 (10.7%)	
pT3	206 (49.3%)	52 (52.0%)	154 (48.4%)	
pT4a	155 (37.1%)	34 (34.0%)	121 (38.1%)	
N stage				0.980 [b]
pN0	207 (49.2%)	50 (49.5%)	157 (49.1%)	
pN1	31 (7.4%)	7 (6.9%)	24 (7.5%)	
pN2a	24 (5.7%)	5 (5.0%)	19 (5.9%)	
pN2b	29 (6.9%)	8. (7.9%)	21 (6.6%)	
pN2c	12 (2.9%)	2 (2.0%)	10 (3.1%)	
pN3b	118 (28.0%)	29 (28.7%)	89 (27.8%)	
No. op positive nodes, median, range	1 (0-20)	0 (0-10)	1 (0-20)	0.869 [c]
Extracapsullar spread	139 (33.0%)	34 (33.7%)	105 (32.8%)	0.874 [b]
Synchronous tumour	35 (8.3%)	9 (8.9%)	26 (8.1%)	0.797 [b]
Location of synchronous tumour				0.029 [b]
Larynx	6 (17.1%)	1 (11.1%)	5 (19.2%)	
Hypopharynx	3 (8.6%)	2 (22.2%)	1 (3.8%)	
Oropharynx	6 (17.1%)	1 (11.1%)	5 (19.2%)	
Oral cavity	3 (8.6%)	3 (33.3%)	0 (0)	
Esophagus	1 (2.9%)	0 (0)	1 (3.8%)	
Thyroid gland	6 (17.1%)	0 (0)	6 (23.1%)	
Skin of head and neck	3 (8.6%)	0 (0)	3 (11.5%)	
Lungs	5 (14.3%)	1 (11.1%)	4 (15.4%)	
Conjunctiva	1 (2.9%)	0 (0)	1 (3.8%)	
Lymph nodes	1 (2.9%)	1 (11.1%)	0 (0)	

Table S2. Potential risk factors for PCF associated with the disease by univariate analysis for all patients ([a]T-test, [b]Chi square test, [c]Mann-Whitney U test)

Risk factor associated with the surgical treatment	Overall	PCF	Without PCF	P value
**All patients**	**422**	**101**	**321**	
Dyspnoea management				
Tracheostomy	116 (27.5%)	21 (20.8%)	95 (29.6%)	0.084 [b]
Debulking	25 (5.9%)	3 (3.0%)	22 (6.9%)	0.149 [b]
Surgery of primary tumour				
TL + partial hypopharyngectomy	164 (38.9%)	46 (45.5%)	118 (36.8%)	0.114 [b]
TL + total hypopharyngectomy	6 (1.4%)	2 (2.0%)	4 (1.2%)	0.587 [b]
Neck dissection	346 (82.0%)	79 (78.2%)	267 (83.2%)	0.258 [b]
Unilateral	39 (9.2%)	9 (8.9%)	30 (9.3%)	0.895 [b]
Bilateral	308 (73.0%)	70 (69.3%)	238 (74.1%)	0.340 [b]
Surgical margin status				
R0	397 (95.0%)	93 (94.9%)	304 (95.0%)	0.968 [b]
R1	21 (5.0%)	5 (5.1%)	16 (5.0%)	0.968 [b]
Reconstruction				
epiglottoplasty	54 (12.8%)	21 (20.8%)	33 (10.3%)	0.006 [b]
Regional/microvascular	50 (11.8%)	20 (19.8%)	30 (9.3%)	0.005 [b]
Type regional/microvascular reconstruction				0.009 [b]
PM	37 (8.8%)	13 (12.9%)	24 (7.5%)	
SCAIF	2 (0.5%)	1 (1.0%)	1 (0.3%)	
Radial forearm	3 (0.7%)	2 (2.0%)	1 (0.3%)	
Radial forearm-hybrid	3 (0.7%)	3 (3.0%)	0 (0)	
ALT	4 (0.9%)	1 (1.0%)	3 (0.9%)	
Gastric pull-up	1 (0.2%)	0 (0)	1 (0.3%)	
Duration of surgery (h), mean, range	4.8 (1.25-13.00)	5.1 (1.25-13.00)	6.4 (1.25-11.00)	
Surgeons				0.238 [b]
Surgeon 1	67 (15.9%)	20 (19.8%)	47 (14.6%)	
Surgeon 2	45 (10.7%)	14 (13.9%)	31 (9.7%)	
Surgeon 3	5 (1.2%)	2 (2.0%)	3 (0.9%)	
Surgeon 4	55 (13.0%)	10 (9.9%)	45 (14.0%)	
Surgeon 5	52 (12.3%)	13 (12.9%)	39 (12.1%)	
Surgeon 6	68 (16.1%)	9 (8.9%)	59 (18.4%)	
Surgeon 7	54 (12.8%)	11 (10.9%)	43 (13.4%)	
Surgeon 8	6 (1.4%)	2 (2.0%)	4 (1.2%)	
Surgeon 9	37 (8.8%)	13 (12.9%)	24 (7.5%)	
Surgeon 10	3 (0.7%)	0 (0)	3 (0.9%)	
Surgeon 11	30 (7.1%)	7 (6.9%)	23 (7.2%)	
Blood transfusion	99 (23.5%)	31 (30.7%)	68 (21.2%)	0.049 [b]
Antibiotic prophylaxis	421 (99.8%)	101 (100.0%)	320 (99.7%)	0.578 [b]

Type of antibiotic prophylaxis				0.025 [b]
None	1 (0.2%)	0 (0)	1 (0.3%)	
amoxicillin/clavulanate	268 (63.5%)	50 (49,5%)	218 (67.9%)	
clindamycin	141 (33.4%)	49 (48.5%)	92 (28.7%)	
other regimens	12 (2.9%)	2 (2.0%)	10 (3.1%)	
Duration of antibiotic prophylaxis	7.57 (0-48)	9.28 (1-48)	7.03 (0-35)	<0.001 [a]
Primary speech prosthesis	21 (5.8%)	2 (2.2%)	19 (7.1%)	0.090 [b]

Table S3. Potential risk factors for PCF associated with the surgical treatment (TL) by univariate analysis for all patients ([a]T-test, [b]Chi square test, [c]Mann-Whitney U test)

Risk factor associated with the postoperative period	Overall	PCF	Without PCF	P value
**All patients**	**422**	**101**	**321**	
Surgical wound infection	121 (28.7%)	76 (75.2%)	45 (14.0%)	<0.001 [b]
Postoperative laboratory values				
Haemoglobin (g/L), mean, range	113.44, (65-157)	112.63, (82-157)	113.69, (65-151)	0.940 [a]
Albumin	32.78, (19-55)	32.32, (23-44)	32.93, (19-55)	0.157 [a]
Proteins	55.04, (27-80)	54.40, (36-77)	55.24, (27-80)	0.470 [a]
Difference between preoperative and postoperative laboratory values				
Haemoglobin (g/L), mean, range	22.43, (0-50)	28.85, (0-50)	20.48, (0-75)	0.015 [a]
Albumin	8.54, (0-35)	8.86, (0-22)	8.44, (0-35)	0.047 [a]
Proteins	13.89, (0-43)	14.58, (0-32)	13.68, (0-43)	0.137 [a]

Table S4. Potential risk factors for PCF associated with the postoperative course by univariate analysis for all patients ([a]T-test, [b]Chi square test, [c]Mann-Whitney U test)
